# Tubeimoside I induces accumulation of impaired autophagolysosome against cervical cancer cells by both initiating autophagy and inhibiting lysosomal function

**DOI:** 10.1038/s41419-018-1151-3

**Published:** 2018-11-02

**Authors:** Xuping Feng, Jing Zhou, Jingyi Li, Xueyan Hou, Longhao Li, Yongmin Chen, Shuyue Fu, Li Zhou, Changlong Li, Yunlong Lei

**Affiliations:** 1 Department of Biochemistry and Molecular Biology, and Molecular Medicine and Cancer Research Center, Chongqing Medical University, Chongqing, 400016 P.R. China; 2 State Key Laboratory of Biotherapy and Cancer Center, West China Hospital, and West China School of Basic Medical Sciences & Forensic Medicine, Sichuan University, and Collaborative Innovation Center for Biotherapy, Chengdu, 610041 P.R. China; 3 School of Biological Sciences and Technology, Chengdu Medical College, Chengdu, 610083 P.R. China; 4 School of pharmacy, Xinxiang Medical University, Xinxiang, 453003 P. R. China; 5 Department of Oncology, The First Affiliated Hospital of Chongqing Medical University, Chongqing, 400016 P. R. China; 6 Department of Neurology, The Affiliated Hospital of Hainan Medical College, Hainan, 570102 P.R. China; 7 West China School of Basic Medical Sciences & Forensic Medicine, Sichuan University, Chengdu, 610041 P.R. China

## Abstract

Cervical cancer is one of the most aggressive human cancers with poor prognosis due to constant chemoresistance and repeated relapse. Tubeimoside I (TBM) has been identified as a potent antitumor agent that inhibits cancer cell proliferation by triggering apoptosis and inducing cell cycle arrest. Nevertheless, the detailed mechanism remains unclear and needs to be further elucidated, especially in cervical cancer. In this study, we found that TBM could induce proliferation inhibition and cell death in cervical cancer cells both in vitro and in vivo. Further results demonstrated that treatment with TBM could induce autophagosome accumulation, which was important to TBM against cervical cancer cells. Mechanism studies showed that TBM increased autophagosome by two pathways: First, TBM could initiate autophagy by activating AMPK that would lead to stabilization of the Beclin1-Vps34 complex via dissociating Bcl-2 from Beclin1; Second, TBM could impair lysosomal cathepsin activity and block autophagic flux, leading to accumulation of impaired autophagolysosomes. In line with this, inhibition of autophagy initiation attenuated TBM-induced cell death, whereas autophagic flux inhibition could exacerbated the cytotoxic activity of TBM in cervical cancer cells. Strikingly, as a novel lethal impaired autophagolysosome inducer, TBM might enhance the therapeutic effects of chemotherapeutic drugs towards cervical cancer, such as cisplatin and paclitaxel. Together, our study provides new insights into the molecular mechanisms of TBM in the antitumor therapy, and establishes potential applications of TBM for cervical cancer treatment in clinic.

## Introduction

With 500,000 incident cases and 260,000 deaths annually, cervical cancer has been implicated one of the most common cancers worldwide^1,2^. Primary preventions and early treatment of precancerous lesions have sharply declined the incidence rate in most developed countries; however, the morbidity and mortality remain high in some low-income countries^3,4^. In addition, the primary methods for cervical cancer treatment such as surgery, radiotherapy and adjuvant chemotherapy, have greatly improved the carcinoma survival rate^5,6^. Nonetheless, increasing radioresistance or chemoresistance, repeated relapse and tumor metastasis limit the treatment efficacy, highlighting the urgency of developing novel and reliable therapeutic strategies.

Autophagy is a conservative lysosomal degradation pathway during which the intracellular materials are degraded and recycled^7^. Cellular stress events, such as energy limiting, oxidative stress and nutrient deprivation, result in accumulation of damaged or toxic proteins and organelles that can drive autophagy to sustain cellular homeostasis^8^. The autophagic products, such as amino acids, fatty acids and other small molecules can provide a certain amount of energy and synthetic substrates to maintain adequate energy. Given its “self-digest” function, the role of autophagy in cancer is complex and context-dependent^9^. Autophagy is originally known as a tumor suppressor from the investigation of the tumorigenesis tendency in mice with allelic loss of autophagy-related genes (ATGs). However, increasing studies have implicated its role in tumor promoting by assisting cancer cells survival in stress either from environment or induced by tumor therapy^10,11^. Targeting the autophagy process has been regarded as a novel therapeutic approach^12^. Therefore, development of novel autophagy modulator has rewired a way of cancer treatment.

Tubeimoside I (TBM) is extracted from the tuber of *Bolbostemma paniculatum* (Maxim) Franquet (Cucurbitaceae), a traditional Chinese herb previously used in anti-viral or anti-inflammatory treatment^13^. Recently, growing studies have reported its direct cytotoxity in multiple human cancer cells, characterized by mitochondrial damage, endoplasmic reticulum stress, apoptosis and cell cycle arrest^14–17^. In addition, TBM could sensitize human ovarian cancer cells to cisplatin (CDDP)^18^. TBM has been considered as a promising anticancer agent. However, the underlying mechanism remains unclear and elusive.

In the present study, we found that TBM-treated cervical cancer cells displayed decreased proliferating rate and obvious cell death. TBM also promoted remarkable autophagosome synthesis, resulted from activation of adenosine monophosphate-activated protein kinase (AMPK) signaling. In addition, autophagic flux was blocked in the late stage of autophagic process, eventually leading to impaired autophagolysosomes accumulation and cell death. Moreover, this novel autophagic cell death inducer may enhance the treatment efficacy of chemotherapeutic drugs towards cervical cancer. Our findings suggest that TBM act as a potent autophagy modulator and may provide new insights into therapeutic strategy for cervical cancer.

## Results

### TBM inhibits cervical cancer cells proliferation both in vitro and in vivo

**Fig. 1:**
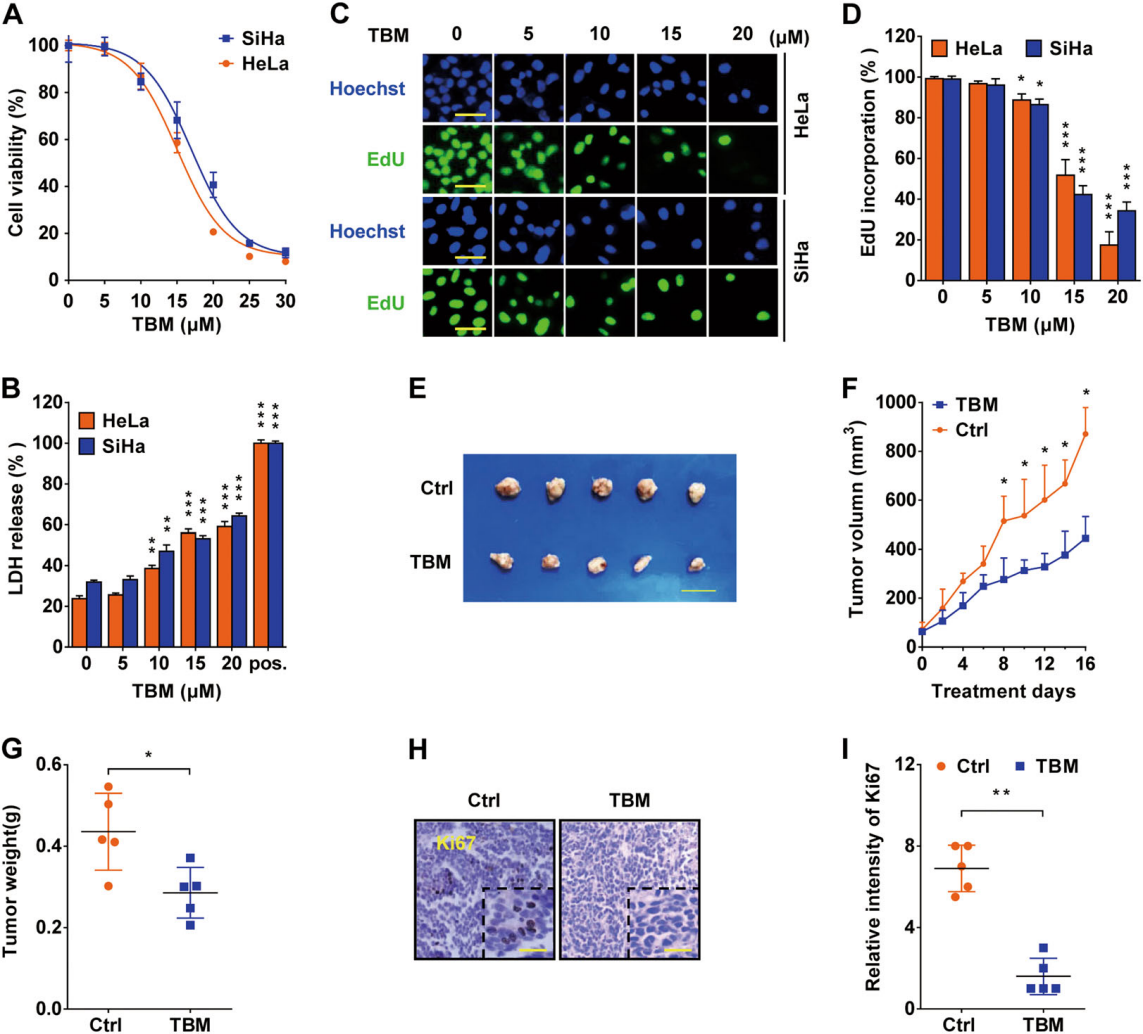
TBM inhibits cervical cancer cells proliferation. **a** Hela and SiHa cells were treated with indicated concentrations of TBM for 24 h. Cell viability was measured by the MTT assay. **b** TBM disrupted cellular membrane integrity as measured by LDH release in the medium. Cells were treated as in (**a**). **c**–**d** Cell proliferation of HeLa and SiHa cells were measured by EdU labeling. Cells were treated as in (**a**). Scale bars: 100μm. **e**–**g** Nude mice bearing HeLa xenograft tumor were treated with 100 μL saline solution (control, *n* = 5) or 3 mg/kg TBM (*n* = 5) daily for 16 days. **e** Tumor tissues were taken and imaged after animals sacrificed. Scale bars: 1 cm. **f** Tumor volumes were monitored every other day and calculated by the length and width. **g** Tumors were weighed immediately once mice were killed. **h**–**i** Tumor tissues were sectioned and subjected to immunohistochemistry for evaluating expression of Ki67. Scale bars: 100 μm. *p < 0.05; **p < 0.01; ***p < 0.001

To identify the role of TBM in cervical cancer, cervical cancer cell lines (HPV18-positive HeLa and HPV16-positive SiHa) were treated with TBM. MTT assay showed that TBM markedly decreased the cervical cancer cells’ viability in a dose-dependent manner (Fig. 1a). LDH release assay also revealed that TBM could damage the integrity of plasma membrane (Fig. 1b). As shown in Supplementary Figure 1, cells exposed to TBM exhibited a significant survival inhibition, as evidenced by the decreased colony numbers. Furthermore, in comparison to controls, a notably lower rate of EdU-postive cells was observed in TBM-treated cells (Figs. 1c, d), indicating the growth inhibitory effect of TBM on cervical cancer cells.

To further define the antitumor effects of TBM in vivo, we established a mouse xenograft model with HeLa cells, following by receiving TBM or saline solution treatment. Tumor size, volume and mass increased dramatically in the vehicle control. In contrast, tumor in TBM-treated mice grew less prominent (Figs. 1e–g). In addition, most of the TBM-treated tumors displayed reduced Ki67 staining (Figs. 1h, i), indicating the decreased proliferating ability in TBM-treated group. Collectively, these results suggest that TBM inhibit proliferation of cervical cancer cells both in vitro and in vivo.

Besides, we found that TBM effectively decreased the cell viability of several cancer cells in a dose-dependent manner, including glioblastoma, breast cancer, hepatocarcinoma, lung cancer and colorectal cancer cells, indicating that TBM is a broad-spectrum antineoplastic agent (Supplementary Figure 2). Unfortunately, TBM could also induce growth inhibition in normal cells (Supplementary Figure 3A and 3B**)**. However, the cytotoxicity of TBM was low when treatment concentration was less than 10 µM, and the cytotoxicity of TBM was equivalent to commonly used anticancer drugs such as cisplatin (CDDP), paclitaxel (PTX), doxorubicin (DOX) and 5-fluorouracil (5-FU) (Supplementary Figure 3C**)**, suggesting that low concentration of TBM is relatively safe and may be benefit for cancer treatment.

### TBM induces apoptosis in cervical cancer cells both in vitro and in vivo

**Fig. 2:**
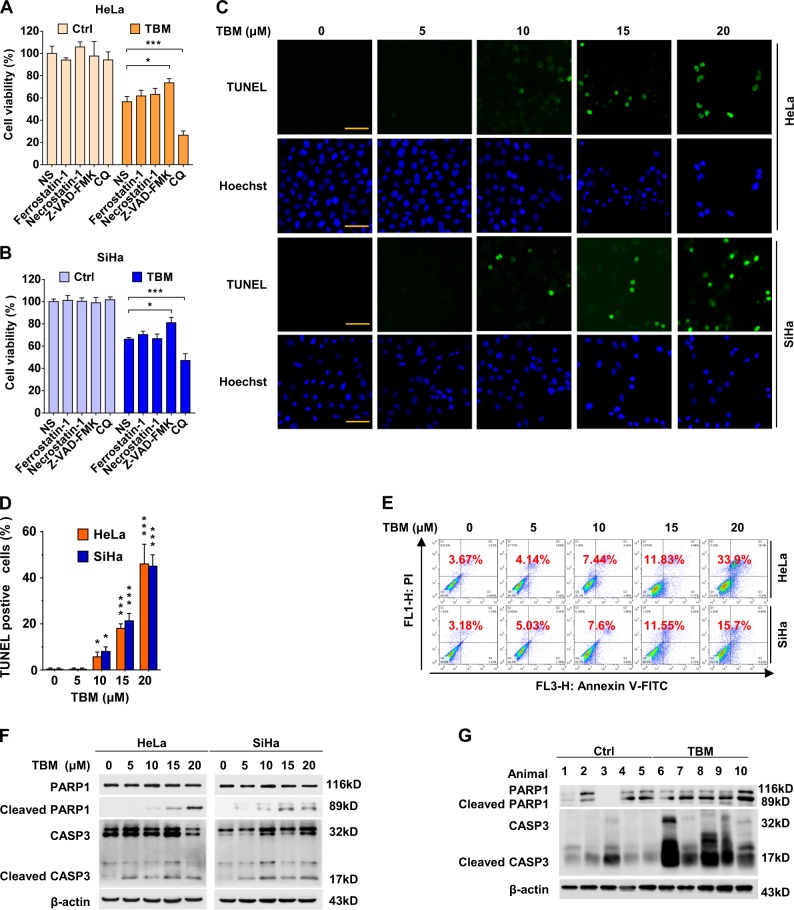
TBM induces apoptosis in cervical cancer cells both in vitro and in vivo. **a**–**b** HeLa and SiHa cells were treated with TBM (15 μM) either alone or combination with specific cell death inhibitors, ferrostatin-1 (5 μM), necrostatin-1 (5 μM), Z-VAD-FMK (10 μM) and CQ (10 μM) for 24 h, and the percentage of cell death was determined by MTT assay. **c**–**d** HeLa and SiHa cells were treated with indicated concentrations of TBM for 24 h, and apoptosis index was determined by TUNEL assay. Scale bars: 100μm. **e** HeLa and SiHa cells were treated with indicated concentrations of TBM for 24 h, and then apoptosis was measured with FACS assay. **f** HeLa and SiHa cells were treated with indicated concentrations of TBM for 24 h, and the expression of cleaved CASP3 and PARP1 was measured with immunoblot. **g** Tumor samples were collected from the mouse xenograft mode, and the expression of cleaved CASP3 and PARP1 was measured with immunoblot. *p < 0.05; ***p < 0.001

To get more insights into the mode of TBM-induced cell death, we treated cervical cancer cells with TBM combination with a series of death inhibitors. As shown in Figs. 2a, b, Z-VAD-FMK, a pan-caspase inhibitor^19^, partially rescued TBM-induced cell death; in contrast, other inhibitors including ferrostatin-1 and necrostatin-1, failed to influence the cell death caused by TBM, in spite of their specific capacities to inhibit ferroptosis^20^ and necroptosis^21^, respectively. This implies that apoptosis might be associated with TBM-induced cervical cancer cell death. Consistently, both TUNEL assay and flow cytometry analysis exerted prominent apoptotic effect in cervical cancer cells (Figs. 2c-e). In addition, cleaved-CASP3 and cleaved-PARP1, one of caspase downstream effectors^22^, were accumulated upon TBM treatment (Fig. 2f). Consistently, TBM also caused apoptosis in cervical cancer in vivo (Fig. 2g). In summary, these data indicate that apoptosis is involved in TBM against cervical cancer both in vitro and in vivo.

### TBM induces autophagosome formation in cervical cancer cells

**Fig. 3:**
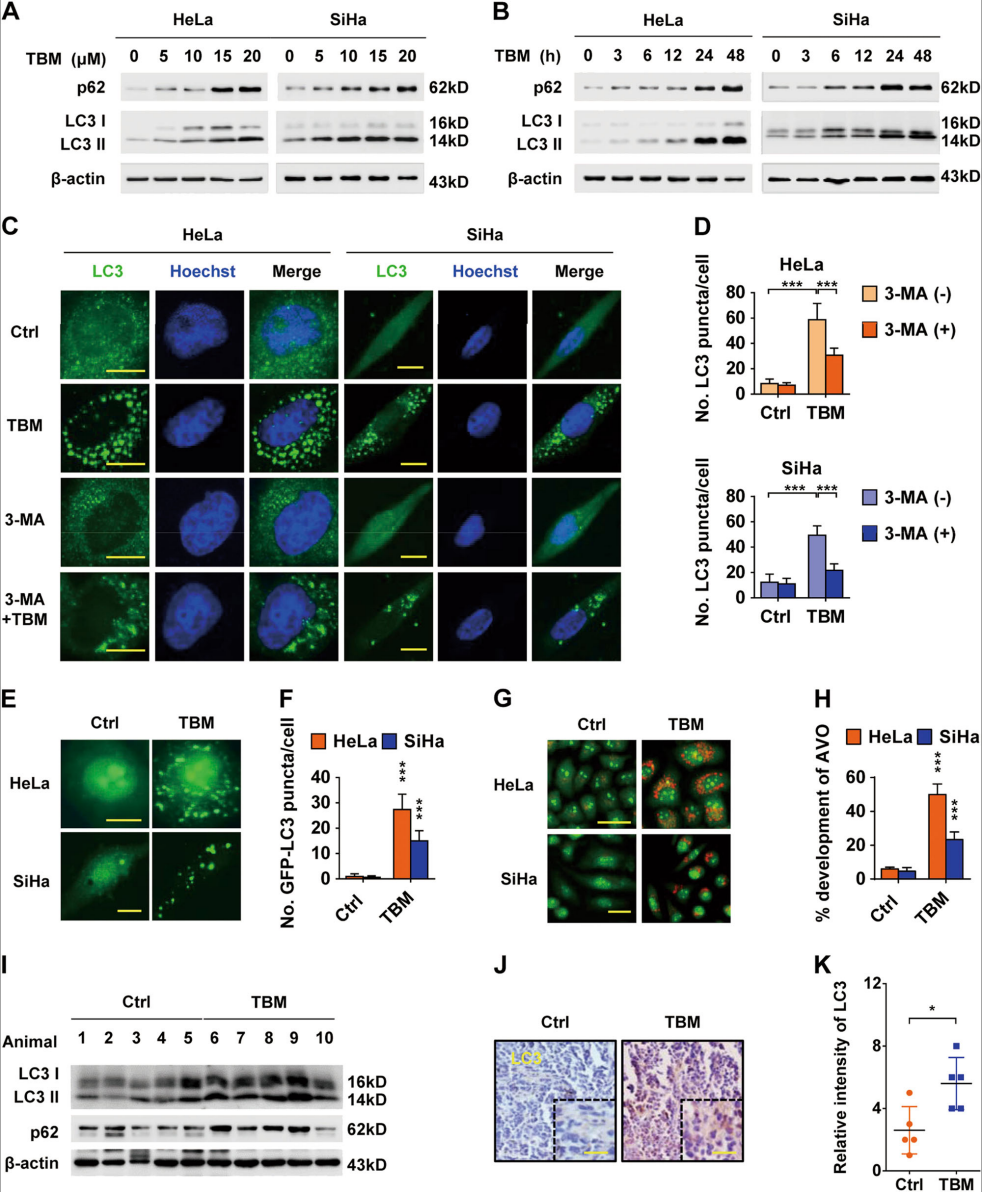
TBM initiates autophagosome formation in cervical cancer cells. **a** Immunoblot analysis of LC3 and p62 in HeLa and SiHa cells treated with indicated concentrations of TBM for 24 h. **b** Immunoblot analysis of LC3 and p62 in HeLa and SiHa cells treated with 15 μM TBM for indicated time. **c** Immunofluorescence analysis of endogenous LC3 puncta formation in TBM-treated HeLa and SiHa cells in present with or without 3-MA for 24 h. Scale bar: 20μm. **d** Graph shows quantification of LC3-positive punctate cells in (**c**). **e** HeLa and SiHa cells were transfected with GFP-LC3 for 48 h, and then treated with 15 μM TBM for another 24 h. GFP-LC3 puncta was visualized by immunofluroescence. Scale bars: 20μm. **f** Graph shows quantification of GFP-LC3-positive punctate cells in (**e**). **g** Acridine orange was used to stain acidic vesicular organelles in HeLa and SiHa cells treated with 15 μM TBM. Scale bars: 50 μm. **h** The total number of acidic vesicular organelles (AVO) per cell in Figure G was quantified by ImageJ software. **i** Tumor tissues were hydrolyzed, and then LC3 and p62 were examined by immunoblot. **j**–**k** Tumor tissues were resectioned and subjected to immunohistochemistry for evaluating expression of LC3 in mouse tumor xenograft. Scale bars: 100 μm. *p < 0.05; ***p < 0.001

Interestingly, as shown in Figs. 2a, b, co-treatment of TBM and CQ could initiate more cell death than TBM alone, indicating that autophagy might be also involved in TBM-induced cell death. To explore the relationship between TBM and autophagy, cervical cancer cells were treated with TBM and then LC3 II conversion, a specific marker of autophagy^23^, was measured. Of note, TBM significantly induced LC3 II accumulation in both dose-dependent and time-dependent manners (Figs. 3a, b). In addition, transmission electron microscopy experiment also showed that the formation of double-membraned autophagic vacuoles was frequently observed in cervical cancer cells treated with TBM (Supplementary Figure 4**)**. Furthermore, both the endogenous LC3 and exogenous GFP-LC3 puncta, representing the number of autophagic vacuoles^23^, were remarkably increased in TBM-treated cells (Figs. 3c–f). Acidic vesicular organelles, which are markers of autophagosomes^24^, were also markedly increased following TBM treatment (Figs. 3g, h), highlighting its capacity in inducing autophagosome accumulation.

To examine whether TBM induces autophagy in vivo, tumor samples were analyzed for LC3 II level. We observed an increase of LC3 II conversion by immunoblot analysis (Fig. 3i). Consistently, TBM-treated xenografts displayed stronger LC3 staining compared with the control group (Figs. 3j, k). In summary, TBM can induce autophagosome accumulation in cervical cancer both in vitro and in vivo. In addition, TBM also induced autophagosome accumulation in various types of cancer cells as evidenced by the LC3 II accumulation (Supplementary Figure 5).

### TBM initiates autophagy by activating AMPK

**Fig. 4:**
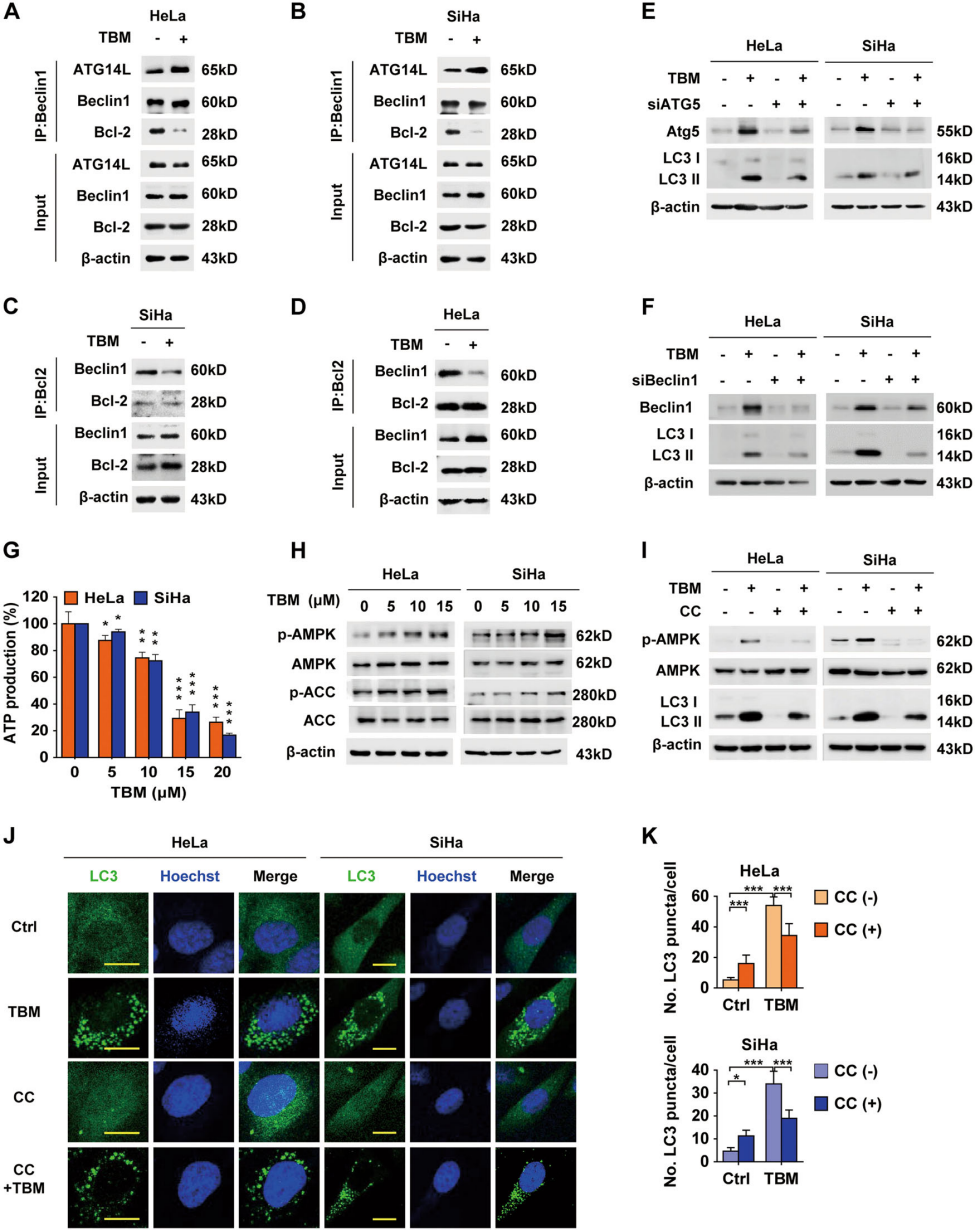
TBM induces an AMPK-dependent autophagosome formation. **a**–**b** Interaction among Beclin1, Atg14L and Bcl-2 in HeLa (**a**) and SiHa (**b**) cells was determined by co-immunoprecipitation assay. **c**–**d** Interaction between Bcl-2 and Beclin1 in HeLa (**c**) and SiHa (**d**) cells was determined by co-immunoprecipitation assay. **e** HeLa and SiHa cells were transfected with siScramble or siATG5 for 48 h, and then treated with 15 μM TBM for another 24 h. Expression of Atg5 and LC3 was examined by immunoblot. **f** HeLa and SiHa cells were transfected with siScramble or siBeclin1 for 48 h, and then treated with 15 μM TBM for another 24 h. Expression of Beclin1 and LC3 was examined by immunoblot. **g** ATP production was detected in cells treated with indicated concentrations of TBM by ATP Assay Kit (Beyotime Biotechnology, S0026). (**h**) Immunoblot analysis AMPK, ACC, AMPK phosphorylation (Thr172) and ACC phosphorylation (Ser79) in cells treated with the indicated concentrations of TBM for 24 h. β-actin was used as the internal control. **i** Cells were treated with TBM in the absence or presence of CC. AMPK, AMPK phosphorylation (Thr172) and LC3 were measured by immunoblot. **j** Cells were treated with TBM in the absence or presence CC for 24 h. LC3 puncta formation was measured by immunofluorescence analysis. Scale bars: 20 μm. **k** Graph shows quantification of LC3-positive puncta in Figure J. *p < 0.05; **p < 0.01; ***p < 0.001

Once autophagy initiated, a series of ATGs will drastically be activated to mediate autophagosome formation. A key event in the autophagy pathway is the activation of the Beclin1-Vps34 complex, which functions in the formation of isolated membrane (also known as the phagophore). Intriguingly, we found that Beclin1 showed a relatively strong interaction with ATG14 and weak binding affinity toward Bcl-2 in TBM-treated cells (Figs. 4a-d), suggesting that TBM could stabilize the Beclin1-Vps34 complex by dissociating Bcl-2 from Beclin1. In addition, treated with TBM combination with 3-MA or wortmannin (WTM), the PtdIns3K inhibitors that selectively block activation of Beclin1-Vps34 complex^25^, obviously decreased LC3 II and LC3 dots formation in cervical cancer cells (Figs. 3c, d; Supplementary Figure 6A and 6B). Furthermore, molecular inhibition of ATG5 or Beclin1 by siRNA revealed a decrease in LC3 II accumulation (Supplementary Table 1, Figs. 4e, f). These results show that TBM can initiate autophagy in vitro.

AMPK and mTOR signaling pathways are two main autophagy-initiating pathways^26^. Considering that TBM could damage the mitochondria and cause the depletion of mitochondrial transmembrane potential (∆Ψm)^27,28^, which subsequently decreased the production of energy in the form of adenosine triphosphate (ATP), we hypothesized that AMPK might play a critical role in TBM-induced autophagy. To confirm this, we firstly measured the ATP levels in HeLa and SiHa cells treated with TBM. As expected, both cells exerted a dose-dependent decrease of ATP production (Fig. 4g). Immunoblot also showed that TBM could activate AMPK and its down-stream protein, ACC, in a dose-dependent manner (Fig. 4h). To further exam the role of AMPK in TBM-induced autophagy, CC, a specific AMPK inhibitor, was used to inactivate AMPK^29^. Of note, CC decreased TBM-induced accumulation of LC3 II and formation of LC3 puncta (Figs. 4i, k). In summary, these data indicate that TBM initiates autophagy by activating AMPK in cervical cancer cells.

### TBM inhibits autophagic flux in cervical cancer cells

**Fig. 5:**
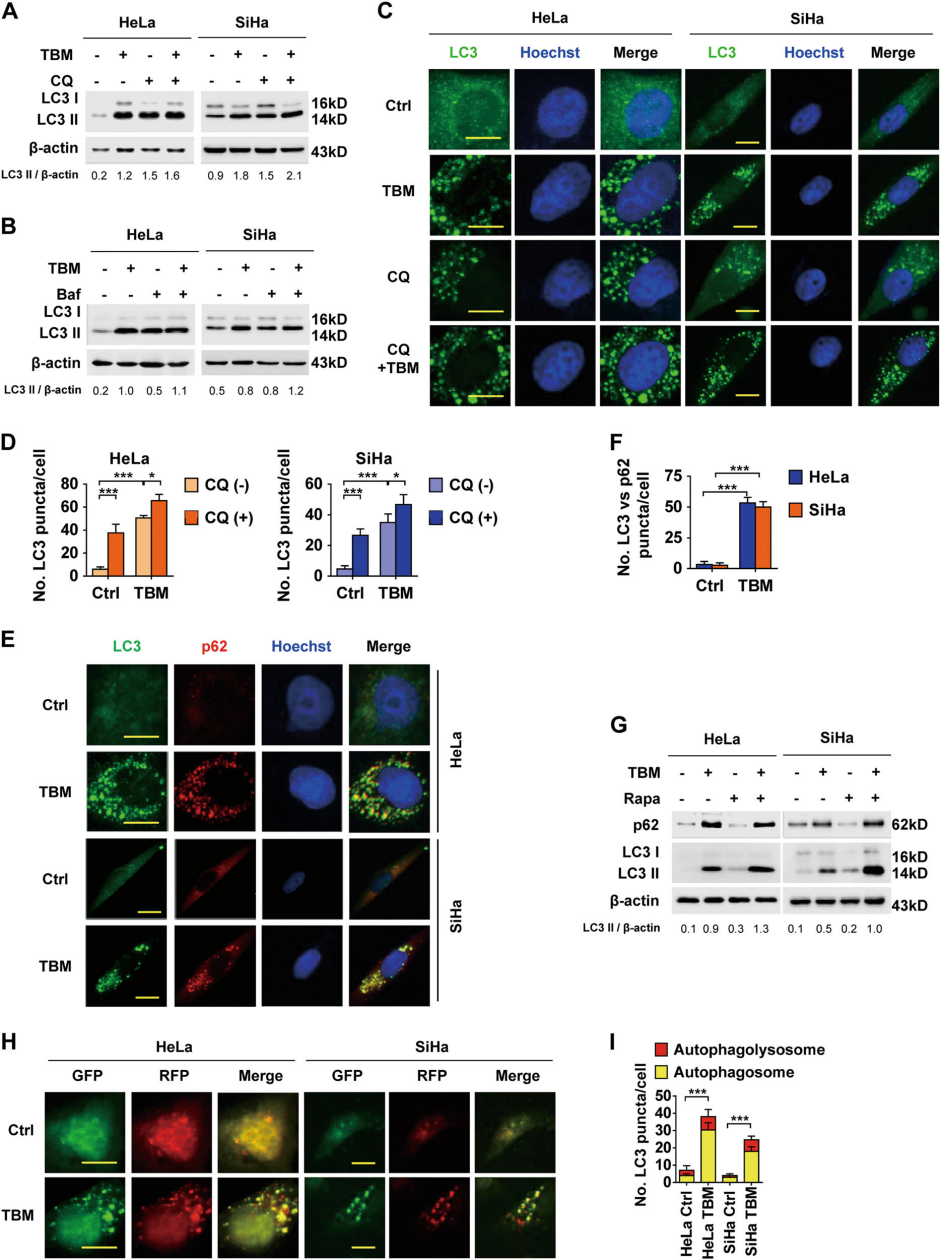
TBM inhibits autophagic flux in cervical cancer cells. **a** HeLa and SiHa cells were treated with TBM in the absence or presence of CQ (10 µM). LC3 was measured with immunoblot. **b** Cells were treated with TBM in the absence or presence of bafilomycin A1 (Baf, 100 nM). LC3 was measured with immunoblot. **c**–**d** HeLa and SiHa cells were treated with TBM in the absence or presence of 10 µM CQ. LC3 puncta formation was measured by immunofluorescence analysis and quantified by ImageJ. **e**–**f** Cells were treated with TBM. LC3 and p62 puncta were measured by immunofluorescence analysis and quantified by ImageJ. **g** HeLa and SiHa cells were treated with TBM in the absence or presence of rapamycin (500 nM). LC3 and p62 expression was measured with immunoblot. The ratio of LC3 II/β-actin was determined with ImageJ software. **h**–**i** Cells were transfected with mCherry-GFP-LC3 for 48 h, and treated with TBM for another 24 h. The formation of autophagosome (mCherry-positive; GFP-positive) and autophagolysosome (mCherry-positive; GFP-negative) was examined and quantified by ImageJ. Scale bars: 20 μm. *p < 0.05; ***p < 0.001

In addition to initiate autophagy, our earlier observations also showed that TBM induced an increase of p62 (Figs. 3a, b, i), a substrate of autophagy which is delivered to the lysosomes for degradation^8^. Enhanced p62 expression can be either associated with increased protein synthesis or due to discontinuity of autophagosome turnover^30^, suggesting that TBM may inhibit autophagic flux. To clarify the underlying mechanism, HeLa and SiHa cells were treated with TBM in combination with Baf A1 (a V-ATPase inhibitor)^31^ or CQ (a lysosomotropic compound)^32^, both of which could block the end stage of autophagy. As shown in Figs. 5a-d, Baf A1 or CQ induced much less further accumulation of LC3 II protein and LC3 dot numbers in TBM-treated cells compared with control cells, supporting the inference that TBM may inhibit autophagic flux. Additionally, TBM treatment resulted in accumulation of p62 dots and increased colocalization with LC3 punta (Figs. 5e, f), which also suggested autophagic flux inhibition. Furthermore, autophagic flux induced by rapamycin could also be impeded by TBM, suggesting TBM-induced autophagic flux inhibition is independent on the mechanisms of TBM-initiated autophagy (Fig. 5g). Finally, tandem mCherry-GFP-LC3 reporter assays also determined TBM-induced autophagic flux inhibition evidenced by that exposure to TBM caused notable formation of yellow fluorescent autophagosomes and moderate increase of red fluorescent autophagolysosomes **(**Figs. 5h, i). In summary, these results show that autophagic flux inhibition plays a role in TBM-induced accumulation of autophagosomes.

### TBM blocks autophagic flux by impairing lysosomal enzyme

The inhibition of autophagic flux could probably be attributed to impaired fusion between autophagosome and lysosome^33^. To determine whether lysosomal trafficking was affected by TBM, we assessed the colocalization of endogenous LC3 with LAMP1, which represents the formation of autophagolysosome^34^. Surprisingly, TBM induced a significant overlap between LC3 and LAMP1 and Pearson’s correlation coefficient equaled about 0.5 (Supplementary Figure 7A and 7B), indicating that TBM induced fusion between autophagosome and lysosome. In line with the results, examination of the endogenous colocalization of LC3 and LysoTraker Red, a specific dye for lysosome labeling, also confirmed that autophagosome-lysosome fusion was not impaired by TBM (Supplementary Figure 7C and 7D).

Next, we detected whether TBM might deregulate the function of lysosome. We first examined the lysosomal pH, as maintaining acidification is essential for lysosomal activity^35^. However, AO staining displayed a significant increase of acidic vesicles in TBM-treated cells (Figs. 3g, h). To further monitor the lysosomal pH, cells treated with TBM was applied with LysoSensor Green DND-189 (pKa~5.2) and analyzed with flow cytometry. As shown in Supplementary Figure 8A, an increasing shift of acidic compartments was observed in cells with TBM treatment, indicating the role of TBM in retaining the acidic environment. Similar result was obtained using LysoTracker Red (Supplementary Figure 8B), which accumulated in acidic lysosomes. These results indicate that TBM-induced autophagic inhibition is not correlated with lysosomal pH alteration.

**Fig. 6:**
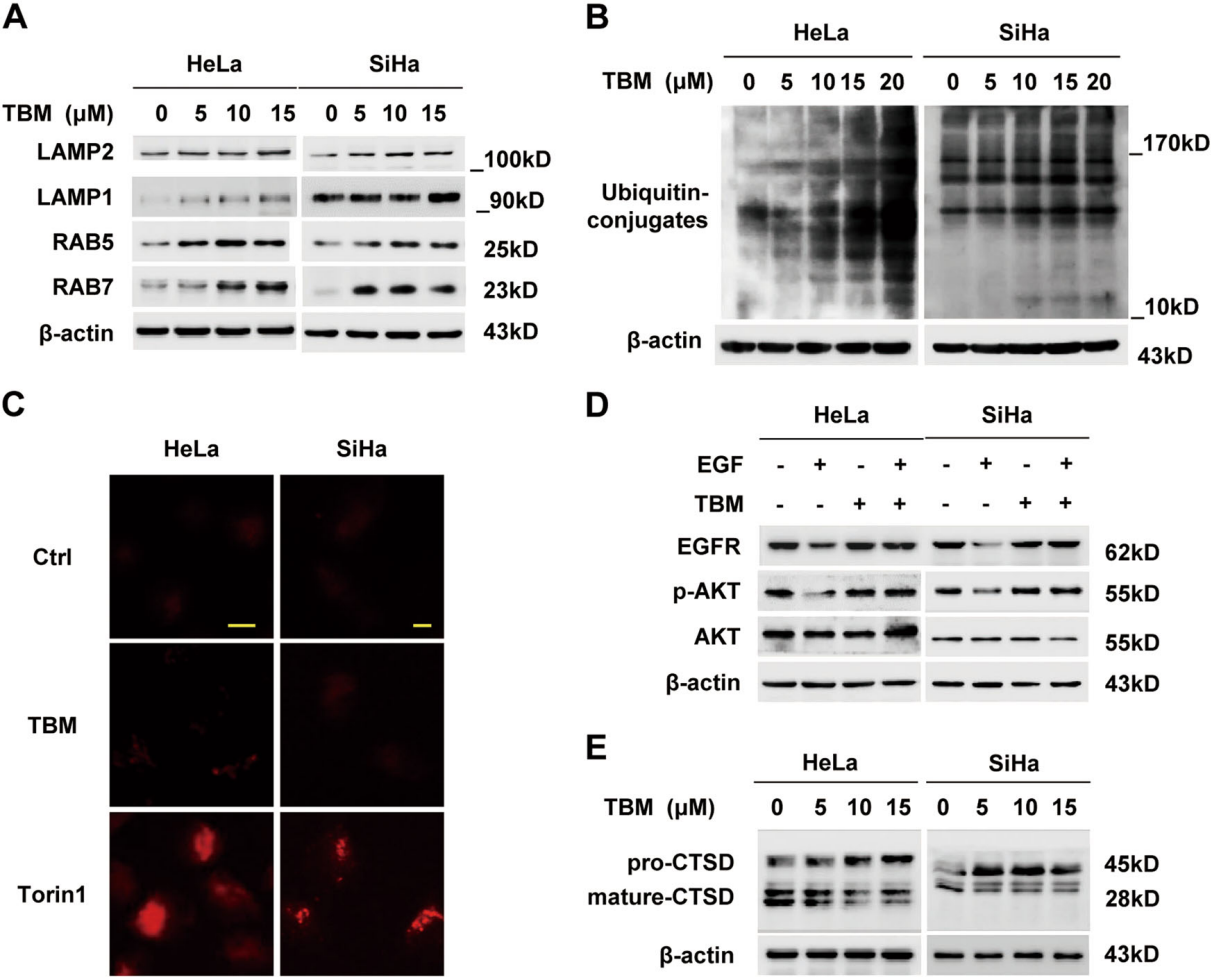
TBM impairs lysosomal hydrolytic activity in cervical cancer cells. **a** Cells were treated with indicated concentrations of TBM for 24 h. The expression of LAMP2, LAMP1, RAB5, and RAB7 in whole cell lysates was determined by immunoblot. **b** Immunoblot of ubiquitin in HeLa and SiHa cells treated with TBM in indicated concentrations for 24 h. **c** Autophagolysosomes stained with DQ-BSA in HeLa and SiHa cells treated with 15 μM TBM for 24 h. Accumulation of fluorescent signal indicated the lysosomal proteolysis of DQ-BSA. Torin1 acted as positive control. Scale bar: 10 μM. **d** HeLa and SiHa cells were starved of serum overnight and treated with EGF (100 nM) in the absence or presence of 15 μM TBM for 2 h. EGFR, AKT and phosphorylation of AKT (Thr 308) were analyzed by immunoblot. **e** Immunoblot analysis of endogenous CTSD in HeLa and SiHa cells treated with 15 μM TBM for 24 h

Now that TBM maintained an adequate pH in cervical cancer cells, we next examined the expression of LAMP1, LAMP2, RAB5 and RAB7, which are critical membrane proteins for lysosome and endosome and are important for lysosomal physiology^35^. Results showed that all proteins exhibited an increase in TBM-treated cells on a dose-dependent manner (Fig. 6a), indicating that the disturbed autophagic flux was not due to the decreased membrane protein expression of lysosomes and endosomes.

In the end, we examined the function of lysosomal enzymes. Accumulating evidence indicated that dysfunction of lysosomal hydrolytic enzymes resulted in an increase of ubiquitinated proteins^36^. As expected, TBM treatment induced accumulation of ubiquitinated proteins in HeLa and SiHa cells (Fig. 6b). Furthermore, DQ-BSA was applied to monitor general endosomal-lysosomal process, during which the red fluorescence of DQ-BSA was supposed to self-quench without proteolytic cleavage^37^. Notably, very little dequenching occurred in TBM-treated cells, indicating the TBM impaired lysosomal enzymes’ activity (Fig. 6c). Similarly, the epidermal growth factor receptor (EGFR) degradation assay was performed in HeLa and SiHa cells. Accordingly, complex formed by EGF and its receptors might be endocytosed, and then transferred to lysosomes for degradation^38^. As shown in Fig. 6d, TBM inhibited EGF-triggered EGFR degradation, verifying the lysosomal dysfunction. In addition, we investigated the capacity of TBM on cathepsin processing in cervical cancer cells. Our data showed that TBM remarkably prevented the maturation of CTSD, resulting in inhibition of lysosomal activity (Fig. 6e). In summary, our data demonstrate that TBM inhibits lysosomal cathepsin activity, thereby leading to accumulation of impaired autophagolysosomes.

### Accumulation of impaired autophagolysosomes contributes to TBM-induced cell death

**Fig. 7:**
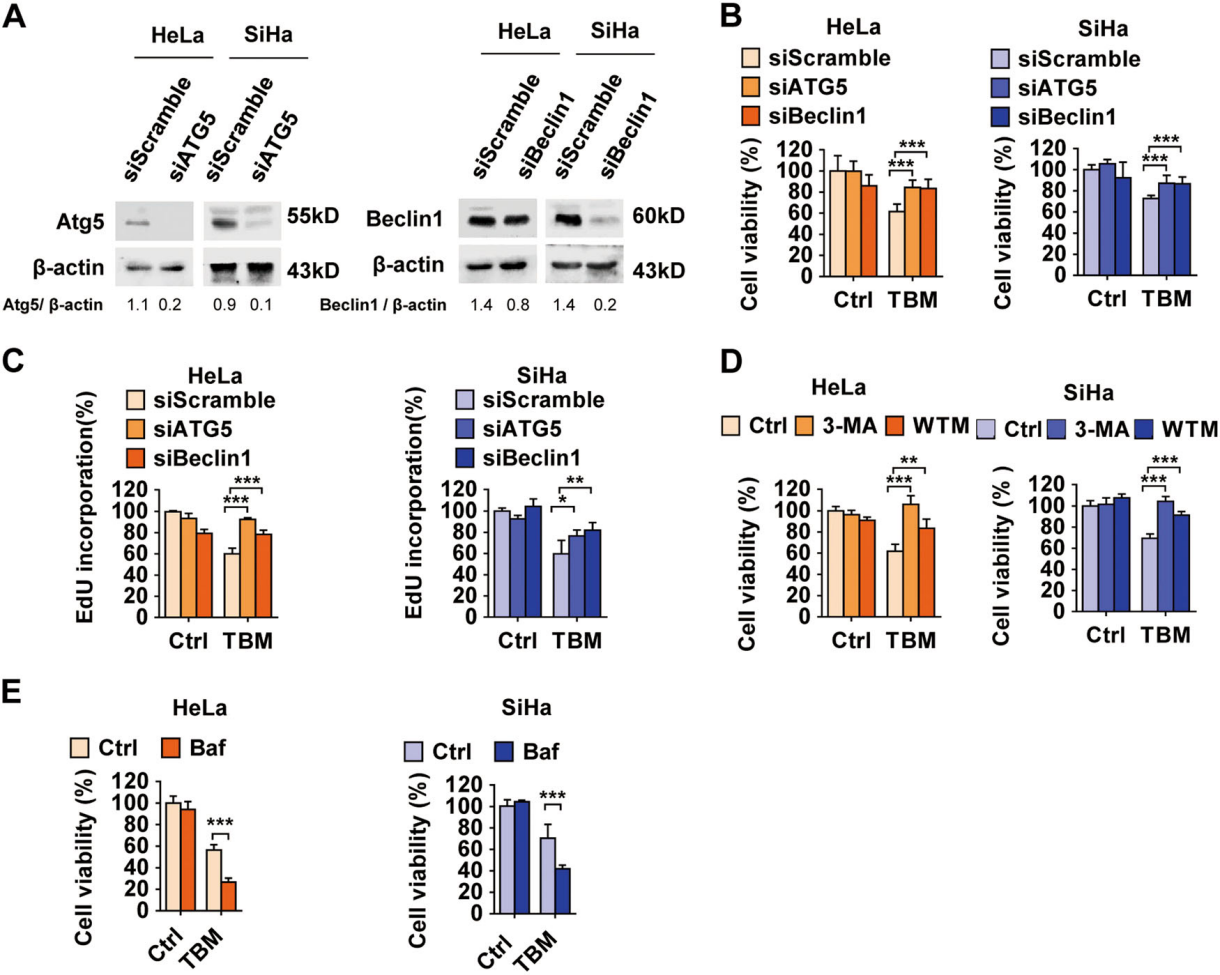
Accumulation of impaired autophagolysosomes is contributed to TBM-induced cell death. **a** Cells were transfected with siScramble, siATG5 or siBeclin1 for 48 h. The expression of Atg5 and Beclin1 was measured by immunoblot. The ratio of Atg5/β-actin and Beclin1/β-actin was determined with ImageJ software. **b**–**c** Cells were transfected with siScramble, siATG5 or siBeclin1 for 48 h, and then treated with 15 μM TBM for another 24 h. **b** Cell viability was detected by MTT assay. **c** Proliferation rate was detected by EdU labeling. **d** Cells were treated with 15 μM TBM in the absence or presence of 3-MA or WTM. Cell viability was determined by MTT assay. **e** Cells were treated with 15 μM TBM in the absence or presence of bafilomycin A1 (Baf). Cell viability was determined by MTT assay. *p < 0.05; ***p < 0.001

Insufficient autophagy, which would cause accumulation of damaged proteins or organelles, may be a disaster to cancer cells and lead to cell death^39^. To investigate the link between autophagy blockade and cell death induced by TBM, we transfected cervical cancer cells with Beclin1 or ATG5 siRNA, followed by TBM treatment. Cell viability was measured with MTT assay and EdU labeling. These data demonstrated that reduction in ATG5 or Beclin1 levels rescued the cell viability caused by TBM (Figs. 7a–c). Furthermore, similar results were obtained by using the autophagic inhibitors, 3-MA or WTM (Fig. 7d). Intriguingly, combination use of TBM with CQ or Baf A1 exacerbated TBM-induced cell death (Figs. 2a, b, 7e). In summary, these data indicate that TBM-induced autophagolysosome accumulation contributes to cell death in cervical cancer cells.

### TBM enhances chemotherapeutic sensitivity through autophagy modulation

**Fig. 8:**
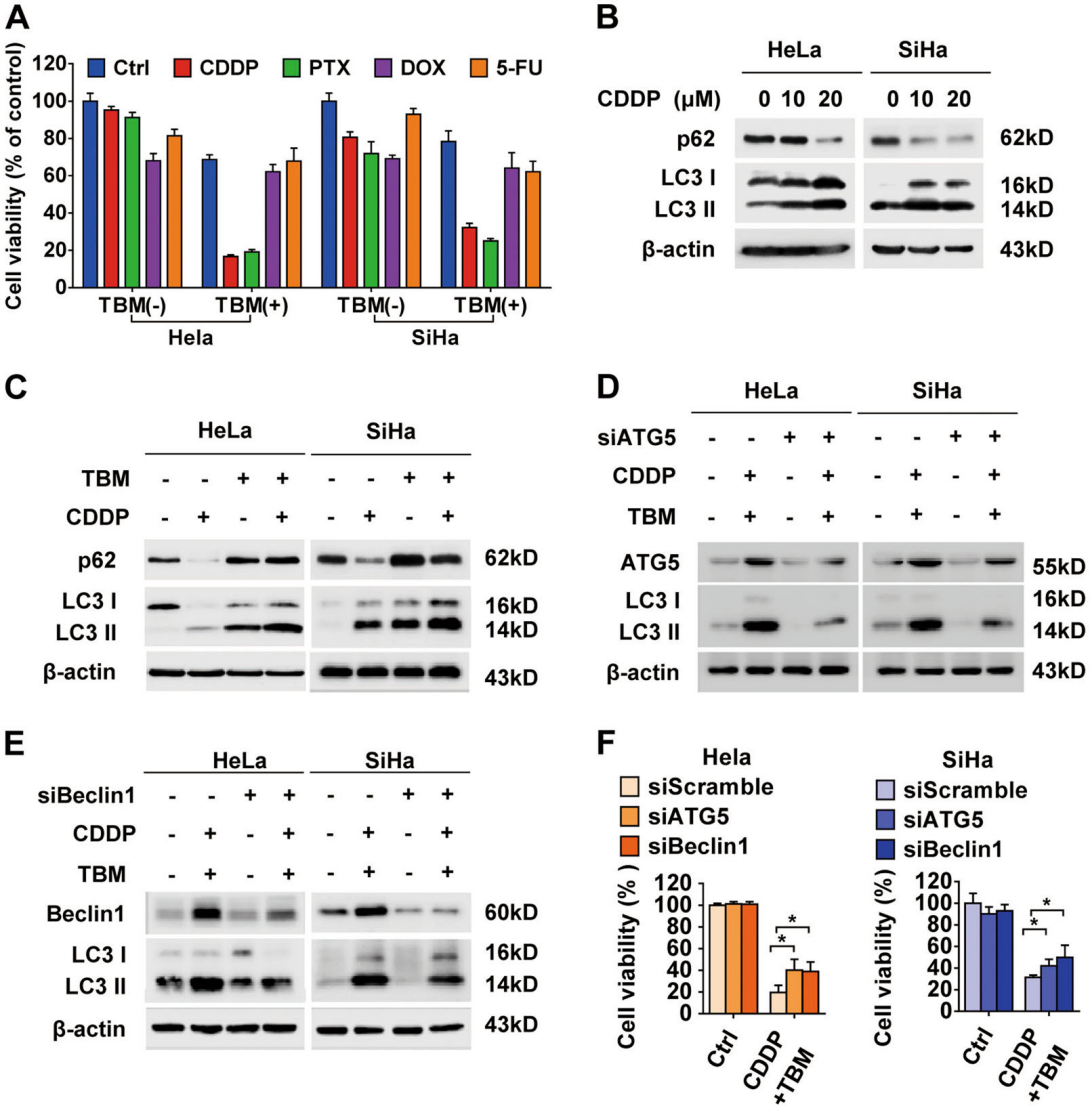
TBM enhances chemotherapeutic sensitivity through autophagy modulation. **a** HeLa and SiHa cells were treated with chemotherapeutic drugs, including cisplatin (CDDP, 10 μM), paclitaxel (PTX, 100 nM), doxorubicin (DOX, 0.25 μM) and 5-fluorouracil (5-FU, 2 μM) in the absence or presence of 10 μM TBM for 24 h and MTT assays were performed to assess cell viability. **b** Cells were treated with CDDP in indicated concentrations for 24 h, and the expression of LC3 and p62 was measured with immunoblot. **c** HeLa and SiHa cells were treated with 10 μM CDDP in the absence or presence of TBM for 24 h, and the expression of LC3 and p62 was measured with immunoblot. **d**–**e** Cells transfected with siScramble, siATG5 or siBeclin1 were co-treated without or with TBM and CDDP, and then subjected to monitor the expression of LC3 II conversion. **f** Cells transfected with siScramble, siATG5, or siBeclin1 were co-treated without or with TBM and CDDP. Cell viability was determined by MTT assay. *p < 0.05

Previous studies have found that autophagy inhibition can enhance the efficacy of chemotherapeutic agents by abolishing the chemoresistance of cancer cells^40,41^. As a specific autophagy modulator, we therefore explored whether TBM would sensitize cervical cancer cells to several chemotherapeutic drugs, including CDDP, PTX, DOX and 5-FU. Co-treatment of HeLa and SiHa cells with TBM and CDDP or PTX in 24 h, resulted in remarkable decrease in cell viability than that of DOX and 5-FU, whereas minimal cytotoxicity was observed when monotherapy was given (Fig. 8a). While co-treatment for 48 and 72 h, TBM treatment combined with DOX or 5-FU caused a more severe increase of cell death (Supplementary Figure 9). Furthermore, in one HPV negative human normal epithelial cell line (HaCat), TBM would significantly enhance the cytotoxicity in DOX-treated cells, but slightly in PTX, 5-FU and CDDP-treated cells, which is different with cancer cells (Supplementary Figure 10**)**. These results suggest that low concentration of TBM, acting as a chemosensitizer or autophagy modulator, is relatively safe in combination treatment with some drugs, or may produce severe side reaction when co-treated with other drugs.

To reveal the underlying mechanism, we focused on the combination between TBM and CDDP, since CDDP is the first line drug in clinical cervical cancer treatment^42^. It has been reported that CDDP might induce a protective autophagy (Fig. 8b), a cause of chemoresistance^42^. As improvement of therapeutic efficacy may result from the defected autophagy induced by TBM, we aimed to examine whether autophagy blockade was involved in the combination strategies. As shown in Fig. 8c, increased LC3 II conversion by CDDP was further augmented in the presence of TBM. Meanwhile, CDDP-induced degradation of p62 was obstructed by TBM, suggesting that CDDP-induced autophagy flux was blocked by TBM and more impaired autophagolysosomes were accumulated. Furthermore, molecularly inhibiting autophagy by transfecting cervical cancer cells with ATG5 or Beclin1 siRNA decreased the LC3 II expression (Figs. 8d, e). Accordingly, in combined treatment, cell proliferation was partially restored by knockdown of ATG5 and Beclin1 (Fig. 8f), indicating that autophagy initiation was involved in TBM-induced accumulation of impaired autophagolysosomes and enhanced cytotoxicity. In summary, these data indicate that TBM may be a potent synergistic application as an autophagy modulator for cancer treatment.

## Discussion

TBM, an ingredient from traditional Chinese herb, has been recently emerged to play a tumor-suppressive role in multiple cancers^17,43–46^. However, the molecular mechanisms have not been clearly determined. Here, we demonstrated that TBM was a potent autophagy modulator. TBM induced an AMPK-dependent autophagy initiation and inhibited autophagic degradation by inhibiting lysosomal proteolysis, leading to autophagolysosomes accumulation in cervical cancer cells. Meanwhile, our findings indicated that accumulation of immature autophagolysosomes by TBM triggered cell death, and enhanced CDDP-mediated cytotoxicity in cervical cancer cells.

Autophagy is a basic catabolic process, which captures unnecessary or dysfunctional cellular components and fuses with lysosomes for degradation^34^. The function of autophagy in cancer therapy is controversial^10,11^. For one hand, autophagy may contribute to cell death;^47,48^ for the other hand, autophagy may play a supportive role in drug resistance^49^. In this study, we demonstrated that TBM could induce biosynthesis of autophagosome, which was subsequently engulfed by the lysosomes to form autophagolysosomes. However, the autophagolysosomes failed to degrade the cargo due to defects of lysosomal function. Inhibition of autophagy initiation restored cell proliferation, while autophagolysosome inhibitors aggravated the cell death incidence, suggesting that TBM functions as a promising antitumor agent by inducing a particular autophagy inhibiting effect.

The AMPK signaling pathway is a well-recognized pathway accounting for autophagy^50^. As one of the major metabolic sensors, AMPK plays an important role in modulation of the autophagy process. Traditionally, AMPK triggers autophagy by either directly activating unc-51 like autophagy activating kinase 1 (ULK1) or indirectly inhibiting mechanistic target of rapamycin (mTOR), both of which eventually lead to activation of Beclin1-Vps34 complex through phosphorylating Beclin1^51^. We here demonstrated that TBM remarkably decreased ATP production, thereby inducing phosphorylation of AMPK and activation of Beclin1-Vps34 complex to initiate autophagy. In addition to initiation of autophagy, increasing studies have revealed the role of AMPK in the late stage of autophagy, such as autophagic proteolysis. For one hand, AMPK can increase the cellular ATP levels to promote autophagic degradation^52^. For the other hand, autophagy flux may also be inhibited with AMPK activation when intracellular nutrition depletion is too excessive, as the autophagy requires adequate amount of ATP to complete this complicated membrane flow-dependent process^53^. Thus, the mechanism of AMPK-mediated autophagy under TBM treatment is complex and remains to be further elucidated.

Lysosome has been considered as the degradation center in most eukaryotic cells^54^. Maintaining lysosomal physiology is essential for autophagy. For instance, the lysosomal acidification, lysosomal trafficking, lysosome fusion with late endosome or autophagosome, and maturation of lysosomal proteases are the most important impacts for lysosomal physiology^55^. In our study, the disruption of lysosomal activity induced by TBM was due to the dysfunction of lysosomal enzyme. In addition, a stale lysosomal function is essential for complete autophagy and cellular homeostasis, especially in tumor cells suffering from stress condition, in contrast, accumulation of autophagolysosomes due to defect lysosomal activity will result cancer cells death^56,57^. It is therefore conceivable that the late stage inhibition of autophagy is contributed to the cytotoxicity of TBM in cervical cancer cells. However, further investigation is needed to be performed on the detailed mechanism of impaired lysosomal proteolysis by TBM.

Recently, the importance of interaction between drug resistance and autophagy in cancer treatment has been widely emerged. Currently, autophagy inhibitors can be classified into early-stage inhibitors (wortmannin, LY294002 and 3-MA) and late-stage inhibitors (CQ, HCQ and Baf A1)^41^. Extensive preclinical studies have demonstrated that autophagy inhibition could enhance the treatment efficacy of chemotherapeutic drugs towards cancer^26,40^, so novel autophagy inhibitors are worthwhile to be exploited. In this study, we found that TBM markedly promoted the sensitivity of CDDP in cervical cancer cells via modulating autophagy. Remarkably, considering that TBM displayed certain antitumor bioactivity, combination of TBM with other chemotherapeutic drugs might be more effective in cancer therapy.

In summary, our study demonstrates that TBM triggers cell death and promotes CDDP sensitivity in cervical cancer by inducing the accumulation of impaired autophagolysosomes. Our findings suggest that TBM is a specific autophagy modulator and could be potentially developed as an adjuvant for further cancer treatment. Our research provides a basis for potential use of TBM in further cancer therapy, especially in cervical cancer treatment.

## Materials and methods

### Cell lines and culture

Human cervical cancer cell lines HeLa and SiHa, glioblastoma cell lines U87 and U251, colorectal cancer cell lines HCT116, HT29 and SW480, hepatocarcinoma cell lines HepG2 and Hep3B, lung cancer cell lines A549 and H1299, human intestinal epithelial cell line HIEC, as well as human lung fibroblasts cells MRC5 were obtained from the American Type Culture Collection (ATCC). Huh7 cell was purchased from the Type Culture Collection of the Chinese Academy of Sciences. Human breast cancer cell lines MCF-7 and MDA-MB-468 were provided by Professor Qiang Yu (Genome Institute of Singapore). Human normal epithelial cell line HaCat cells was provided by Professor Qunying Lei (Fudan University of China). Human fetal normal liver cells LO2 cells were obtained from our lab storage. All cells were cultured in Dulbecco’s modified Eagle’s medium or RPMI1640 (Invitrogen, Carlsbad, CA, USA), supplemented with 10% fetal bovine serum (FBS; Gibco, 10100-147), 10^5^ U/L penicillin, and 100 mg/L streptomycin at 37 °C in an atmosphere containing 5% CO_2_.

### Cell viability measurement

Cells were treated with TBM alone or combination with other drugs. After treatment, 0.5 mg/mL 3-(4, 5-dimethyl-2-thiazolyl)-2, 5-diphenyl-2-H-tetrazolium bromide (MTT) was added and incubated for 4 h. The insoluble formazan product of cells was dissolved in dimethyl sulfoxide after three times washing with phosphate buffered saline (PBS). The optical density (OD) of each culture well was measured by spectrophotometry at 570 nm. The OD value of the control cells was taken as 100% viability. Cellular membrane integrity was assessed with the lactic dehydrogenase (LDH) Release kit (Beyotime), as described previously^58^. Briefly, LDH activity was assayed by adding 100 μL potassium phosphate buffer within 23 mM pyruvate and 0.3 mg/mL β-NADH. Then the conversion of NADH to NAD^+^ was monitored at 340 nm with a spectrophotometer.

### Flow cytometry

Cell apoptosis was analyzed using a PI/Annexin V kit (KeyGEN Biotech). Cells were washed once with PBS, and resuspended in PI//Annexin V solution. At least 20000 live cells were analyzed. The flow cytometry data were collected with FACSCalibur flow cytometer (Becton Dickinson, San Jose, CA, USA), and then analyzed with FlowJo software.

### Tumor xenograft model

The study was subjected to the Institutional Animal Care and Treatment Committee of Sichuan University. Female BALB/c nude mice at 4 weeks of age were obtained from Beijing HFK Bioscience and kept in a sterile environment. HeLa cells (5 × 10^6^) resuspended in 100 μL PBS were injected subcutaneously into the right dorsal region of each mouse, respectively. Seven days after implantation, mice were randomly divided into 2 groups (*n* = 5/group). (1) Control, receiving daily i.p. saline solution (NS); (2) TBM, receiving daily i.p. 3 mg/kg of TBM. Tumor volumes were measured every 2 days determined by measuring the length (l) and the width (w) and calculating the volume (V = l × w^2^/2). After 16 days of treatment, mice were sacrificed. Tumor tissues were isolated and either frozen in liquid nitrogen or fixed in 10% formalin immediately.

### Immunohistochemical analysis

Formalin fixed tissues were embedded by paraffin, and sections were consecutively cut (4μm thickness). The paraffin-sections were dewaxed, rehydrated, and incubated in 3% H_2_O_2_ for 10 min to quench the endogenous peroxidase activity. After antigen retrieval in citrate buffer and incubating with normal rabbit serum for 20 min at 37 °C, the tumor sections were performed with indicated antibodies, following by reaction with diaminobenzidine (Fuzhou Maixin Biotechnology, DAB-0031) and counterstaining with Mayer hematoxylin (Beyotime, C0107). Imaging was obtained with a Leica DM2500 microscope. Immunohistochemical staining was assessed by both the fraction of positive cells (0–100%) and the immunostaining intensity (0-negative, 1-weak, 2-moderate, 3-strong). The final score was calculated by multiplying the fraction score and the intensity score.

### Immunoblot and Immunoprecipitation

Cells were solubilized in lysis buffer and lysates were centrifuged. Supernatant fractions were separated by SDS-PAGE and transferred to polyvinylidene difluoride membranes (EMD Millipore, ISEQ00010). Immunoreactive bands were detected by ECL (EMD Millipore, WBKLS0500). For immunoprecipitations, cell lysates were incubated with 1 μg indicated antibodies overnight at 4 °C, following by addition of protein A-Sepharose beads (40 μL, GE Healthcare) for another 2 h. The samples were analyzed by immunoblot.

### Lysosomal pH measurement

Cells were loaded with 1 mM LysoSensor Green DND-189 (Invitrogen, 10010-023) or LysoTracker Red (C1046) in pre-warm regular medium for 20 min at 37 °C. Then the cells were washed twice with PBS and immediately analyzed by flow cytometry. The flow cytometry data were collected with FACSCalibur flow cytometer (Becton Dickinson, San Jose, CA, USA), and then analyzed with FlowJo software.

### DQ-BSA degradation assay

Cells were plated onto glass coverslips, and incubated with 10 mg/mL DQ-BSA (Thermo Fisher Scientific, D-12051) for 30 minutes. After washing with PBS for three times, the fluorescent signal of DQ-BSA was recorded with a Leica DM2500 microscope.

### Statistical analysis

All data are reported as mean ± SD. Data analysis was performed using Prism 5.0 (Graph-Pad Software, Inc., La Jolla, CA). The statistical significance of the difference between experimental groups in instances of single comparisons was determined using the 2-tailed unpaired Student t test of the means. Comparisons where *p* < 0.05 were deemed significant.

## Electronic supplementary material

Supplementary Materials
