# Supplementary material for: Tubeimoside I induces accumulation of impaired autophagolysosome against cervical cancer cells by both initiating autophagy and inhibiting lysosomal function
Source: Cell Death Dis. 2018 Nov 2;9(11):1117. doi: 10.1038/s41419-018-1151-3 (PMC6214972; doi:10.1038/s41419-018-1151-3)

Supplementary Materials and Methods

Antibodies and reagents

Antibodies: microtubule-associated protein 1 light chain 3 (LC3, PM036), sequestosome 1 (p62, M162-3) and ATG14 (PD026) were purchased from MBL. Lysosomal-associated membrane protein 1 (LAMP1) (sc-20011), LAMP2 (sc-18822), RAB5 (sc-130010), RAB7 (sc-10767), B-cell lymphoma-2 (Bcl-2, sc-492), and β-actin (sc-69879) were purchased from Santa Cruz Biotechnology. ATG5 (12994), Beclin1 (3495), AMPK (5832), p-AMPK (2535), Acetyl-CoA carboxylase (ACC, 3662), p-ACC (11818), protein kinase B (Akt, 4685), p-Akt (4051), cathepsin D (CTSD, 2284), ubiquitin (3933), and poly (ADP-ribose) polymerase 1 (PARP1, 9542) were purchased from Cell Signaling Technology. Caspase 3 (CASP3, MAB4703) was purchased from Millipore. Ki-67 (ab66155) was purchased from Abcam. Goat anti-mouse IgG ($H^{+}L$) secondary antibody, HRP conjugate (31430), and goat anti-rabbit IgG ($H^{+}L$) secondary antibody, HRP conjugate (31460) were purchased from Thermo Fisher Scientific.

Reagents: TBM (BP1415) was purchased from Phytopurify Biotechnology. Ferrostatin-1 (SML0583), necrostatin-1 (N9037), Z-VAD-FMK (V116), 3-methyladenine (3-MA, M9281), wortmannin (WTM, W1628), rapamycin (Rapa, R8781), chloroquine (CQ, C6628) and bafilomycin A1 (Baf, B1793) were purchased from Sigma-Aldrich. Compound C (CC) was purchased from Millipore. Torin 1 (Tor1, S2827) was purchased from Selleck. CDDP (HY-17394), paclitaxel (PTX, HY-B0015), doxorubicin (DOX, HY-15142A) and 5-fluorouracil (5-FU, HY-90006)

were from MedChem Express.

Colony formation assay

Cells were treated with indicated concentrations of TBM for 24 h, and were further cultured for 7 days after medium was changed. Following staining with Giemsa (Sigma-Aldrich, 48900-500ML-F), colonies containing more than 50 cells were counted under a microscope.

Terminal deoxynucleotidyl transferase-mediated nick-end labeling (TUNEL) assays

Cells were plated on glass coverslips in 24-well plates and treated with TBM for 24 h. 4% paraformaldehyde (Sigma) was used to fix the cells, and then TUNEL staining was performed using the DeadEnd Fluorometric TUNEL system (Promega). About two 40x fields of cells were imaged to evaluate the TUNEL-positive cells per coverslip in every independent experiment.

5-ethynyl-2’-deoxyuridine (EdU) labeling assay

The EdU labeling assay was performed in 96-well plate using the EdU Cell Proliferation Assay 5 Kit (Roche). After TBM treatment, 10 μM EdU was added to each well, and the cells were incubated for 12 h at 37 °C. The EdU signaling was determined using a Leica DM2500 microscope.

Immunofluorescence

Cells plated onto glass coverslips were fixed with 4% paraformaldehyde and incubated with 0.1% Triton X-100. The indicated antibody was immunolabeled by incubation at room temperature and then mounted with Prolong Gold with DAPI

(Thermo Fisher Scientific, P36931). Images were obtained with a Leica DM2500 microscope. Five different microscopy images were randomly chosen for quantification analysis. Colocalization is shown in merged as black pixels using the “colocalization” plugin in ImageJ.

Acridine orange (AO) staining

Cells were stained with acridine orange (Invitrogen, A1301) in a final concentration of 5 μg/mL for a period of 10 min (37 °C, 5% CO_2). After washing with PBS 3 times, imaging data was obtained with a Leica DM2500 microscope.

Supplementary figure legends

Supplementary Figure 1. TBM inhibits colony formation in cervical cancer cells. HeLa and SiHa cells were treated with indicated concentrations of TBM for 24 h, and were further cultured for 7 days after medium was changed, with mean colony counts from three independent experiments. **, $p < 0.01$; ***, $p < 0.001$.

Supplementary Figure 2. TBM induces growth inhibition in various cancer cells. Multiple cancer cells were treated with indicated concentrations of TBM for 24 h, including glioblastoma cells (U87 and U251), breast cancer cells (MDA-MB-468 and MCF-7), hepatocarcinoma cells (HepG2, Hep3B and Huh7), lung cancer cells (A549 and H1299) and colorectal cancer cells (HCT116, HT29 and SW480). Cell viability was measured by MTT assay.

Supplementary Figure 3. TBM induces growth inhibition in various normal cell lines. (A) Multiple normal cells were treated with indicated concentrations of TBM

for 24 h, including one HPV negative human normal epithelial cell line (HaCat), human intestinal epithelial cell line (HIEC), human lung fibroblasts cells (MRC5), and human fetal normal liver cells (LO2). Cell viability was measured by MTT assay. **(B)** HaCat cells were treated with indicated concentrations of TBM for 24 h, 48 h and 72 h. Cell viability was measured by MTT assay. **(C)** HaCat cells were treated with 10 or 15 μM TBM, cisplatin (CDDP, 10 μM), paclitaxel (PTX, 100 nM), doxorubicin (DOX, 0.25 μM) or 5-fluorouracil (5-FU, 2 μM) for 24 h. Cell viability was measured by MTT assay.

Supplementary Figure 4. TBM initiates autophagosome formation in cervical cancer cells. Cells were treated with 15 μM TBM, and then autophagy was measured by transmission electron microscopy. Scale bars: 2 μm.

Supplementary Figure 5. TBM induces autophagy in various cancer cells. Multiple cancer cells were treated with 15 μM TBM for 24 h, including glioblastoma cells (U87 and U251), breast cancer cells (MDA-MB-468 and MCF-7), hepatocarcinoma cells (HepG2, Hep3B and Huh7), lung cancer cells (A549 and H1299) and colorectal cancer cells (HCT116, HT29 and SW480). LC3 expression was determined by immunoblot.

Supplementary Figure 6. TBM induces autophagy in cervical cancer cells. **(A)** Cells were treated with 15 μM TBM in the absence or presence of 5 nM 3-MA, and then LC3 expression was examined by immunoblot. **(B)** Cells were treated with 15 μM TBM in the absence or presence 500 nM WTM, and then LC3 expression was examined by immunoblot.

Supplementary Figure 7. TBM does not inhibit autophagosome-lysosome fusion. (A) Colocalization analysis of LC3 (green) and LAMP1 (red) in HeLa and SiHa cells treated with 15 μM TBM for 24 h, respectively. **(B)** The intensity profiles for both fluorescence channels of the white line positioned in (A) were measured using the Plot Profile function in the ImageJ software. **(C)** Colocalization analysis of LC3 (green) and LysoTracker Red (red) in HeLa and SiHa cells treated with 15 μM TBM for 24 h, respectively. **(D)** The intensity profiles for both fluorescence channels of the white line positioned in (C) were measured using the Plot Profile function in the ImageJ software. Scale bars: 20 μm.

Supplementary Figure 8. TBM does not impair lysosomal pH. (A) Cells were treated with 15 μM TBM for 24 h. The cells were incubated with LysoSensor Green DND-189 for 20 min at 37 °C and measured with flow symmetry. **(B)** FACS analysis of LysoTracker Red after treatment with 15 μM TBM for 24 h.

Supplementaey Figure 9. TBM enhances chemotherapeutic sensitivity in cervical cancer cells. (A and B) HeLa (A) and SiHa (B) cells were treated with doxorubicin (DOX, 0.25 μM) in the absence or presence of 10 μM TBM for 24 h, 48 h and 72 h. MTT assays were performed to assess cell viability. **(C and D)** HeLa (C) and SiHa (D) cells were treated with 5-fluorouracil (5-FU, 2 μM) in the absence or presence of 10 μM TBM for 24 h, 48 h and 72 h. MTT assays were performed to assess cell viability.

Supplementary Figure 10. The cytotoxicity of chemotherapeutic drugs to normal epithelial cell is increased when co-treatment with TBM. HaCat cells were treated with chemotherapeutic drugs, including cisplatin (CDDP, 10 μM), paclitaxel (PTX,

100 nM), doxorubicin (DOX, 0.25 μM) and 5-fluorouracil (5-FU, 2 μM) in the absence or presence of 10 μM TBM for 24 h and MTT assays were performed to assess cell viability.

Supplementary Table 1. List of Small Interference RNA Sequences

Gene name	siRNA sense sequences
BECLIN 1	5'-AAGAUAGUGGCAGAAAAUCTT-'3
ATG5	5'-GACGUUGGUAACUGACAAATT-'3
NC	5'-UUCUCCGAACGUGUCACGUUU-'3

Supplementary Figure 1. TBM inhibits colony formation in cervical cancer cells.

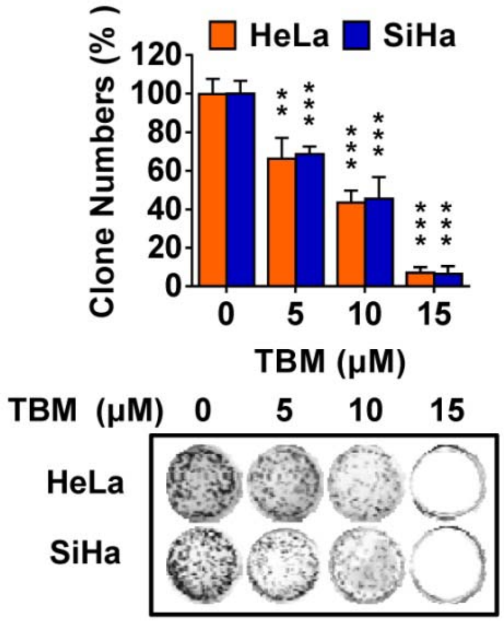

Supplementary Figure 2. TBM induces growth inhibition in various cancer cells.

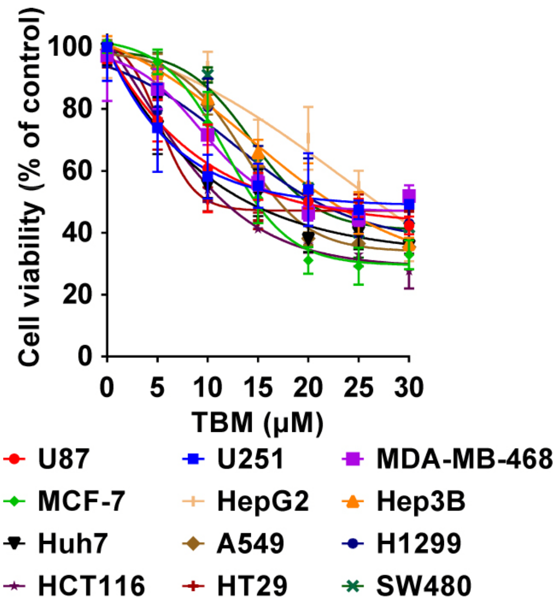

Supplementary Figure 3. TBM induces growth inhibition in various normal cell lines.

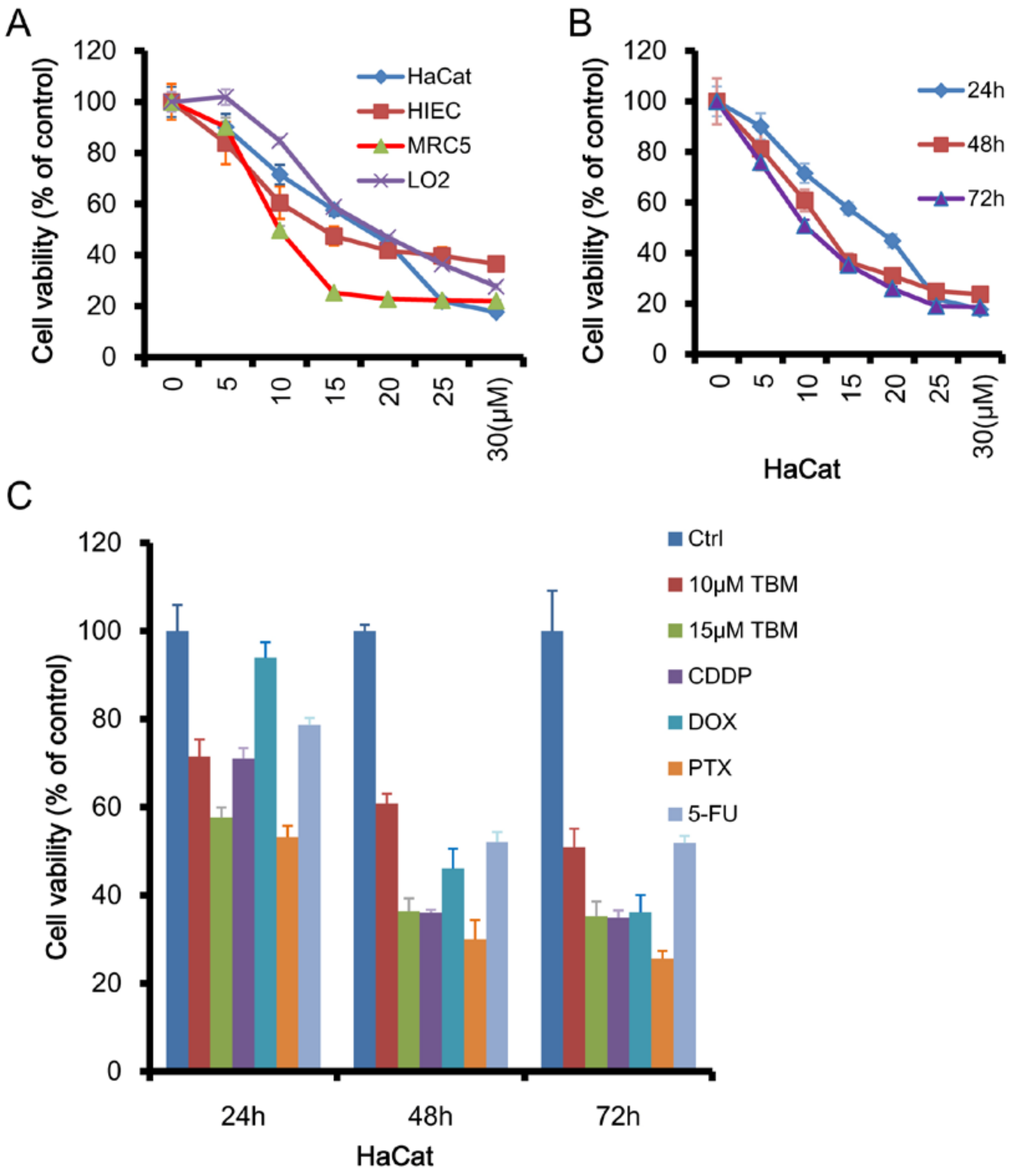

Supplementary Figure 4. TBM initiates autophagosome formation in cervical cancer cells.

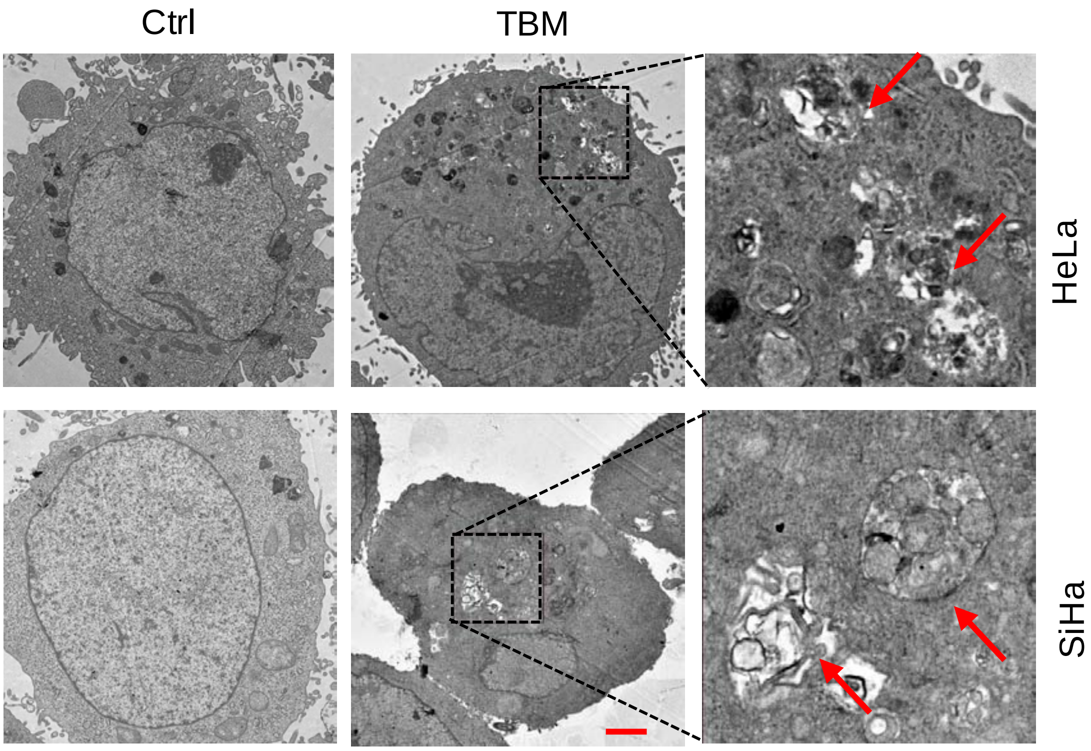

Supplementary Figure 5. TBM induces autophagy in various cancer cells.

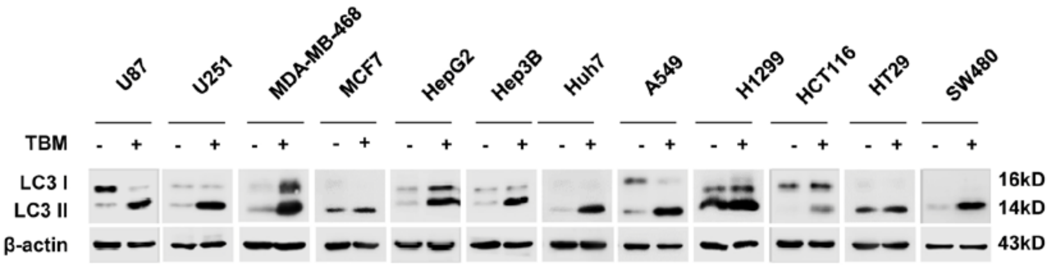

Supplementary Figure 6. TBM induces autophagy in cervical cancer cells.

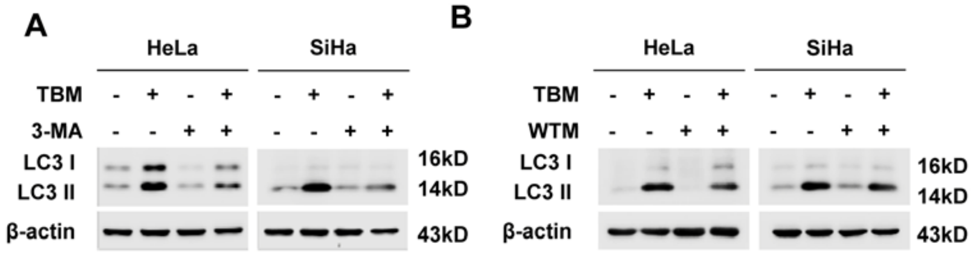

Supplementary Figure 7. TBM does not inhibit autophagosome-lysosome fusion.

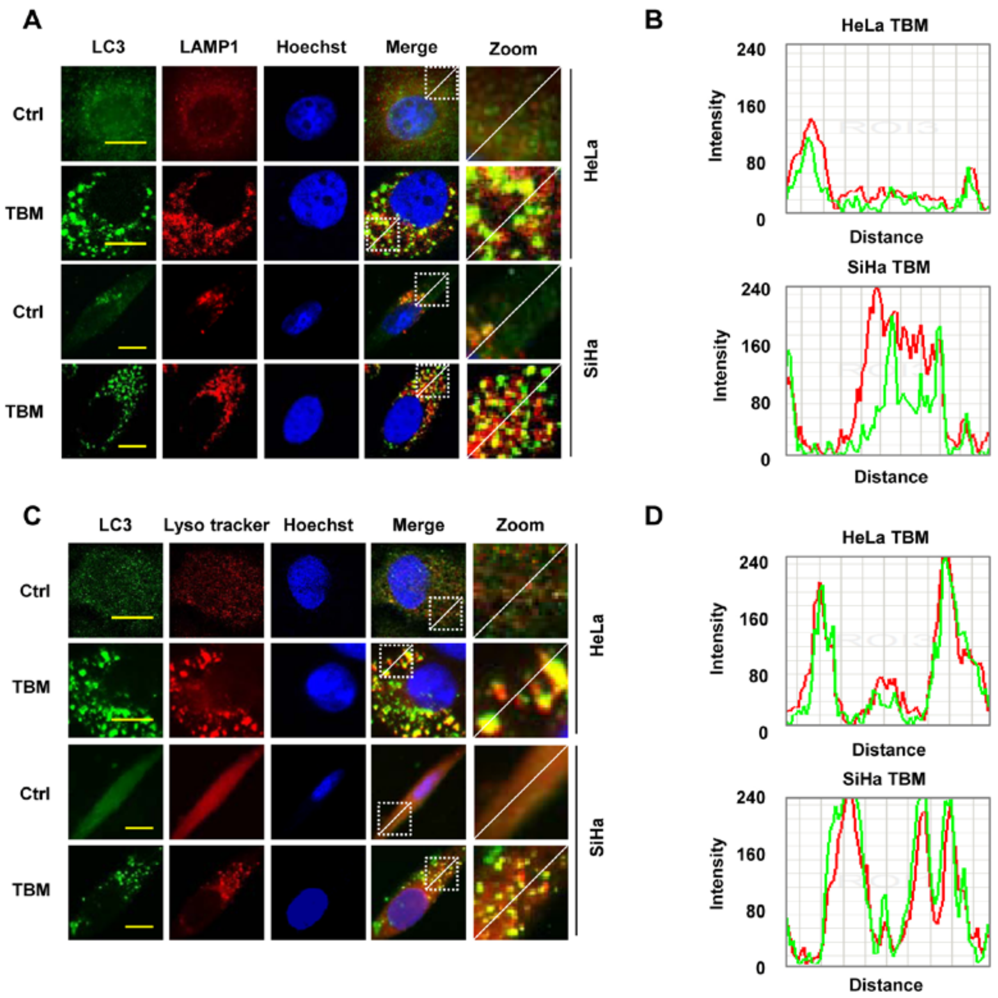

Supplementary Figure 8. TBM does not impair lysosomal pH.

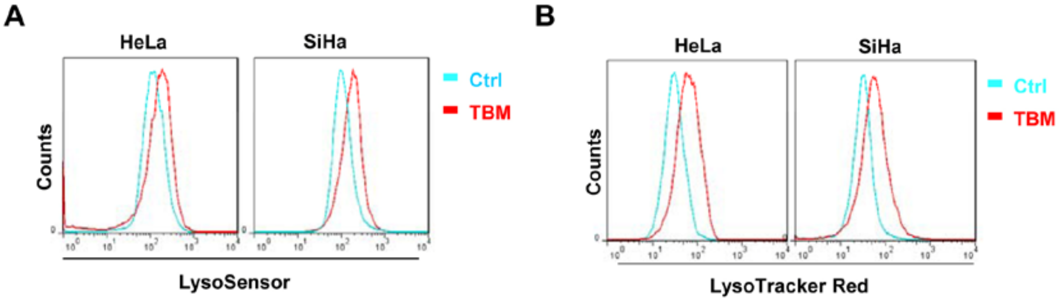

Supplementary Figure 9. TBM enhances chemotherapeutic sensitivity in cervical cancer cells.

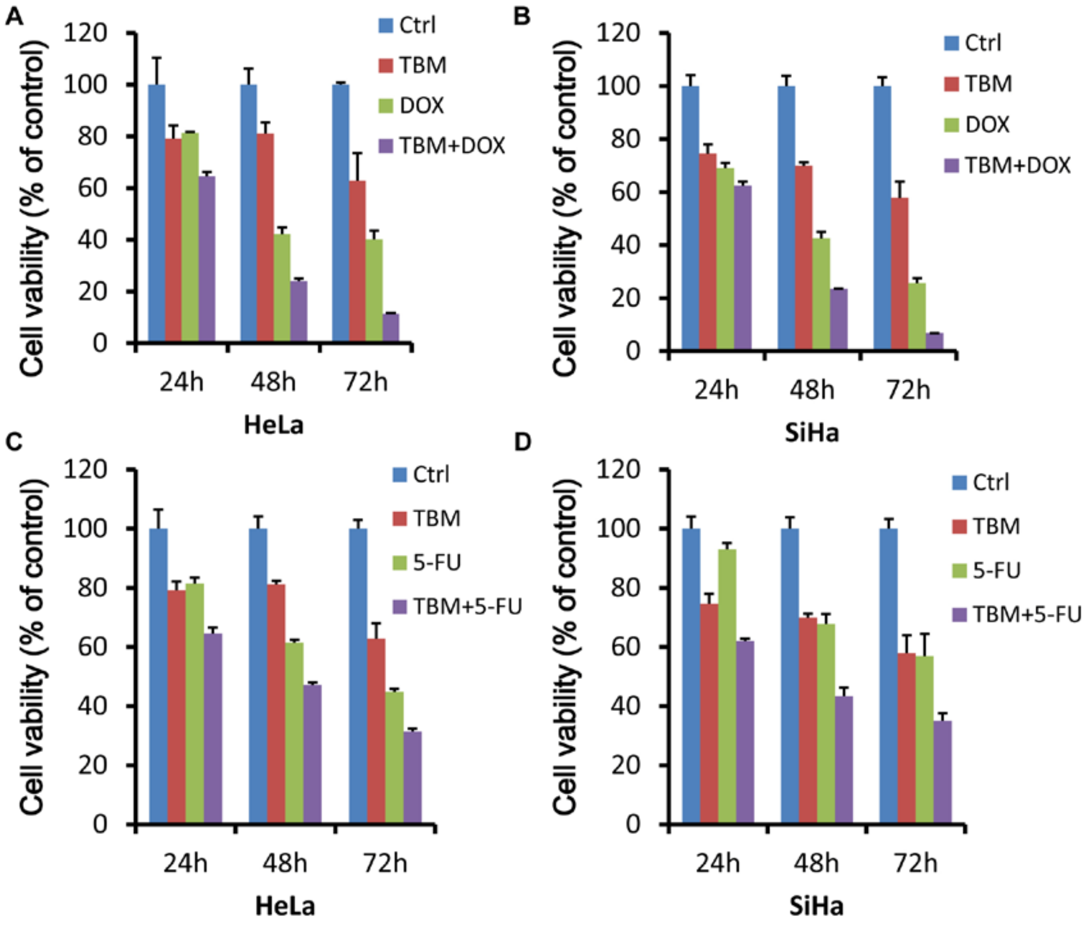

Supplementary Figure 10. The cytotoxicity of chemotherapeutic drugs to normal epithelial cell is increased when co-treatment with TBM.

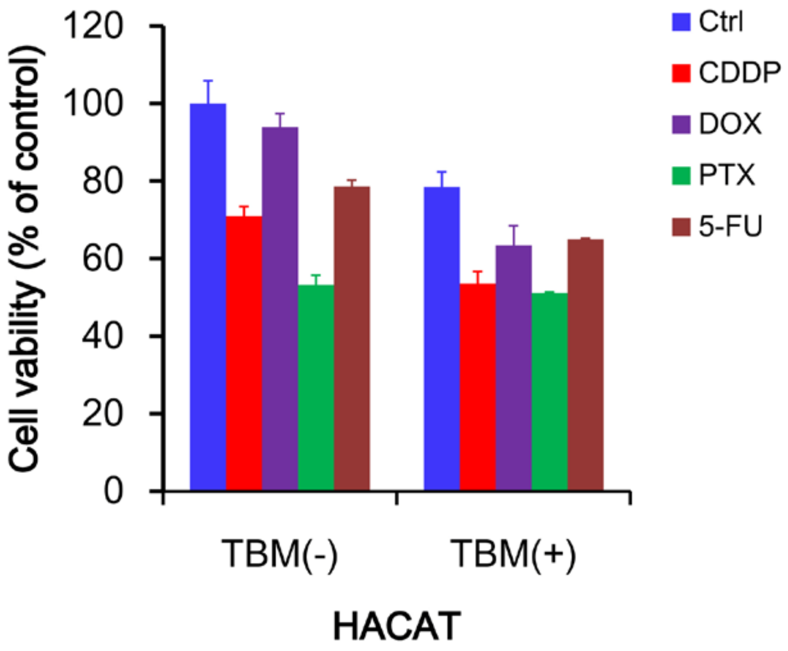

Supplement: Supplementary file 1 — Supplementary Materials [file 41419_2018_1151_MOESM1_ESM.pdf]
